# Social media and scientific research are complementary—YouTube and shrikes as a case study

**DOI:** 10.1007/s00114-017-1470-8

**Published:** 2017-05-24

**Authors:** Łukasz Dylewski, Peter Mikula, Piotr Tryjanowski, Federico Morelli, Reuven Yosef

**Affiliations:** 1 Institute of Zoology, Poznań University of Life Sciences, Wojska Polskiego 71C, 60-625 Poznań, Poland; 2 Department of Zoology, Faculty of Science, Charles University, Viničná 7, 128 43 Praha, Czech Republic; 3 Faculty of Environmental Sciences, Department of Applied Geoinformatics and Spatial Planning, Czech University of Life Sciences Prague, Kamýcká 129, 165 00 Prague, Czech Republic; 4 Faculty of Biological Sciences, University of Zielona Góra, Prof. Szafrana St. 1, 65-516 Zielona Góra, Poland; 5 Ben Gurion University of the Negev–Eilat Campus, P.O. Box 272, 88000 Eilat, Israel

**Keywords:** Social media, Behaviour, Citizen science limits, Shrikes

## Abstract

Fascination with animals and their behaviour is one the most prominent patterns persisting in all human cultures. During the last decades, however, technological development and public access to the Internet have increased the speed and the extent of information sharing at an unprecedented rate, in some cases even challenging the traditional methods used in science. In order to understand the extent of this influence, we focused on the behaviour of shrikes. Shrikes are an enigmatic group of songbirds with a unique behaviour of impaling prey. We employed an extensive Internet search on YouTube (YT), a very popular and increasingly important source of information worldwide, for videos recording shrikes. Our analyses revealed that the number of shrike videos on YT is strongly positively correlated with classical knowledge on shrikes from books and scientific articles. Our results also suggest that in some cases YT may provide an alternative source of information on shrike ecology and behaviour. YT videos may thus provide new insights into the study of certain species or subjects and help identify gaps in ecological studies, especially in poorly studied species.

**Electronic supplementary material:**

The online version of this article (doi:10.1007/s00114-017-1470-8) contains supplementary material, which is available to authorized users.

## Introduction

Since the dawn of human history, cultures have been intrigued by the behaviour of animals (Aelian 2011). Through observations of animal behaviour, the primitive man attempted to immortalise what he observed (Marini et al. 2010; Valenzuela et al. 2015). Examples include animal motifs in temples and tombs, depictions of birds and mammals that accompany ancient idols and gods (Houlihan 1986; Russell and During 2006; Pande and Pande 2016) and that to date influence human thought, superstitious beliefs and daily practices (e.g. Reddy and Yosef 2016). People possess an innate tendency to seek connections with nature (Manfredo 2008). Some behaviour or appearance of animals may trigger a positive emotional response (Wilson 1989). Even today, we are surrounded by animal motifs in everyday life, such as on notes, coins, stamps, company logos, commercial brands, what can be useful and applied for the conservation of biodiversity (Clemmons and Buchholz 1997; Caro et al. 2012). A new approach to nature conservation is the concept of digital conservation, which aims to use the new technological achievements for the protection and monitoring of wildlife (Arts et al. 2015; van der Wal and Arts 2015). The primary approach of digital conservation is open to the collaboration of scientific researchers, non-governmental organisation and citizen science in nature conservation (Arts et al. 2015).

Technological development and public access to the Internet during the last decades have opened the temporal and spatial boundaries and made the world a global village with a rapid flow of information where electronic media has rapidly increased the accessibility and immediacy of information (Valcanis 2011). Since the emergence of technological innovations, such as smartphones and drones, people can at any time share information and post photos and short videos from almost anywhere in the world. For instance, online watching of birds in nest boxes may be important in the context of conducting research but also in education and public outreach (Zárybnická et al. 2017).

Social media may thus play an important role in nature conservation (Cooper et al. 2007; Newman et al. 2012; Chapron 2015; Di Minin et al. 2015; Saito et al. 2015). Citizen science initiative where people can input data into a dedicated website could increase the knowledge about many animal groups, helping protection and monitoring programs (Silvertown 2009). Indeed, many data from social media (e.g. Twitter, Google, YouTube, iNaturalist, Flickr) and Google Earth have been used in actual scientific research (e.g. Visser et al. 2014; Daume 2016; Dyderski et al. 2016; Leighton et al. 2016; Mikula and Tryjanowski 2016; Mikula et al. 2016; Mori et al. 2017).

In addition to websites created for citizen science initiative for a certain issue related to nature conservation (e.g. Bumblebee Conservation Trust, https://bumblebeeconservation.org/get-involved/surveys/beewatch; Blogging Birds, http://redkite.abdn.ac.uk/; HerpMapper, https://www.herpmapper.org/), there remain the unresolved issues of amateur videos about animals posted on YouTube (YT; http://www.youtube.com). YT, with more than a billion users worldwide (https://www.youtube.com/yt/press/statistics.html), has become a very popular and rich source of information during the past decade. YT activities can be divided into two major groups: commercial and educational (Burgess and Green 2013). YT educational materials have been shown to be important not only in third world countries but also in developed countries where it has become an active part of the educational syllabus (Burke and Snyder 2008). Adaptation of YT into our daily lives including neurosurgery (Pereira et al. 2016), infectious diseases (Nagpal et al. 2015), health education and lifestyle (Konstantinidis et al. 2013; Knight et al. 2015) and treatment of medical procedures (Garg et al. 2015) have rapidly become a new and more efficient transmitter of knowledge globally where bandwidth access allows. Through greater access to cameras and mobile phones with video recording capabilities, YT represents a novel tool that facilitates rapid accumulation of data from shared recordings, including those depicting animal behaviour (e.g. Yosef and McPherson 2016).

We chose to study the effect of YT source on true shrikes (*Lanius* spp.), because of our in-depth knowledge and publication history of this family of birds with its unique behaviour of impaling prey (e.g. Yosef and Pinshow 2005; Morelli et al. 2015). This resulted in their discovery by naturalists very early in the publication process (Buffon 1800). Although the behaviour itself was not always properly understood, especially while humans still tended to anthropomorphise and compare animal behaviour to that of humans, this has kept the shrikes at the forefront of scientific research through the ages even by the most eminent of scientists such as Konrad Lorenz (e.g. Lorenz and von Saint Paul 1968). Hence, allowing that we are well versed in the scientific literature pertaining to the shrikes, we tried to understand its relationship with shrike videos on YT and the role of YT videos as a potential source of an alternative knowledge about this bird group. In many shrike species, for which we lack even basic information on their ecology and biology, especially those whose geographic distribution is outside North America and Europe, online sources can represent important sources of alternative information (Harris and Franklin 2000; Panov 2011). Unusual coloration, the mystical facial mask, unique behaviour and proximity to human habitation of the shrikes easily attract the attention of scientists, as well as that of the general public, and hence leading to relatively large amounts of footage being uploaded to YT. In this paper, we aim to address the following parameters: how many shrike videos are on YT, what is their spatial distribution and what types of behaviour they recorded.

## Materials and methods

### Data collection

A search for all available shrike videos was conducted on YT from 2005 to 2015. We searched YT using the scientific and common name of all 29 true shrike species. Only videos where shrikes were observed in their natural environment were included in further analysis. Altogether, we collected 1022 video recordings from 58 countries. On average, video length was 72 s (±2.2 SD), time online since uploading 1204 days (±22.3 SD), number of comments per video 0.74 (±0.11 SD) and the number of views per video 505 (±123.47 SD). More details are included in Table S1. Additionally, we collected data on the country where the video was recorded; in 292 (28.6%) videos, we were unable to determine the country of origin. Furthermore, we collected data characterising each individual bird on video including species identification and recorded behaviour. The behavioural data were classified into eight major categories: (1) feeding, (2) breeding, (3) courtship, (4) defence, (5) singing, (6) comfort activities, (7) relation to humans and (8) others. The category ‘feeding’ included subcategories such as foraging, attack on prey, prey holding, prey decapitation, impaling, drinking water and hovering. The category ‘breeding’ represents all data linked to shrike nesting biology, including brooding, egg hatching, juvenile feeding by parents and fledglings. ‘Courtship’ included videos where courtship behaviour and/or copulation were recorded. Behaviour where shrikes are actively defending themselves against real or potential predators (e.g. human) by mobbing behaviour and defence behaviour we grouped under ‘defence’. ‘Singing’ behaviour included all activities connected with the production of any vocalisation. It included videos where shrikes are presenting a species-specific song, imitating vocalisations of other species or calling. ‘Comfort activities’ included self-dusting, body and beak cleaning, scratching, preening, feather pulling, defecation and pellet casting. The category ‘relation to humans’ included eating from a human hand. In the category ‘other behaviour’, we clumped together video recordings of shrike movements including body and tail wagging, flicking of the tail, moving, looking around and flying where it was not possible to distinguish between aforementioned behaviour types.

### Statistical analyses and data visualisation

We compared the data from YT with the publication record available on shrikes from three different sources: 2047 publications cited in two specific shrike books (Harris and Franklin 2000; Panov 2011), 3965 publications in Web of Science (WoS) and database of 2588 publication lists from the International Shrike Working Group (ISWG; *Lanius* bibliography). We used the Spearman correlation to examine the relationship between the number of shrike videos on YT and number of publications for each shrike species (separately for books, WoS and ISWG). Similarly, we compared the proportion of the human population with an Internet connection and smartphone users to a number of shrike videos by country (for details, see Fig. S1). Chi-square tests were used to compare the proportion of shrike behaviour recorded for eight categories to world region, except videos we were not able to determine the country of origin. All analyses were conducted in R 3.1.3 (R Core Team 2015). The figures were generated with the ggplot2 package (Wickham 2009). The maps were generated with GIS software (ArcGIS 10.1) (ESRI 2012) with the geographical background using data available under the Open Database Licence (‘©OpenStreetMap and contributors’; cartography licenced as CC-BY-SA; http://www.openstreetmap.org/copyright).

## Results

### Relationship between shrike videos and shrike literature

**Fig. 1:**
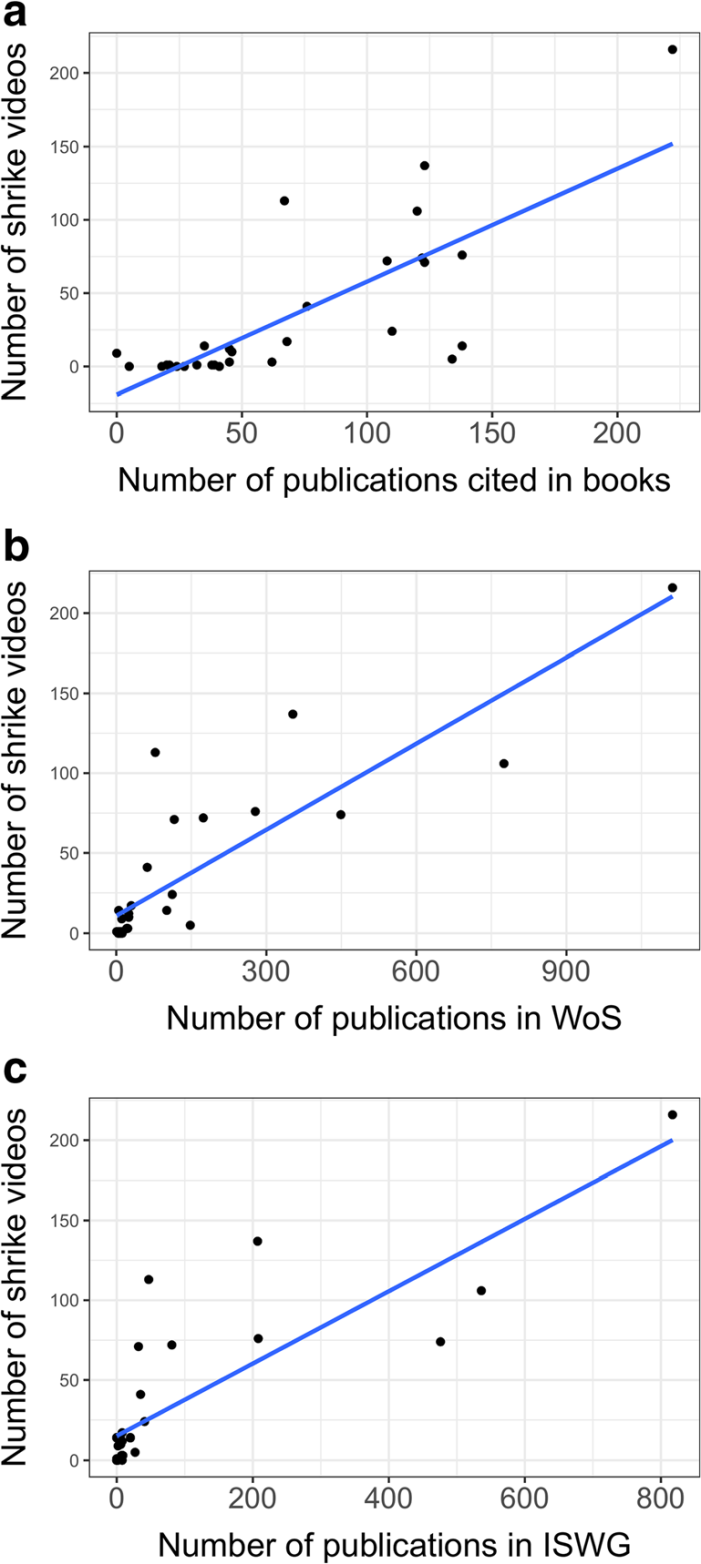
Correlation plots between the number of publications on shrikes and the number of shrike videos on YT for each shrike species

We found YT videos for 24 out of 29 extant species of shrikes. For five shrike species, we did not find any video on YT (*Lanius newtoni*, *Lanius humeralis*, *Lanius dorsalis*, *Lanius gubernator*, *Lanius souzae*), and for another five species (*Lanius mackinnonis*, *Lanius cabanisi*, *Lanius somalicus*, *Lanius excubitoroides* and *Lanius validirostris*), we were able to find only one video. The largest number of videos was available for *Lanius collurio* (21%, *n* = 216 videos), *Lanius senator* (13.5%, *n* = 137), *Lanius bucephalus* (11%, *n* = 113) and *Lanius excubitor* (10.4%, *n* = 106). The number of shrike videos on YT was positively correlated with the number of publications cited in shrike books (*r* = 0.791, *p* = 0.001, Fig. 1a), the number of publications in WoS (*r* = 0.848, *p* = 0.001, Fig. 1b) and the ISWG bibliography (*r* = 0.851, *p* = 0.001, Fig. 1c) for each shrike species.

### Shrike videos and Internet and smartphone users

**Fig. 2:**
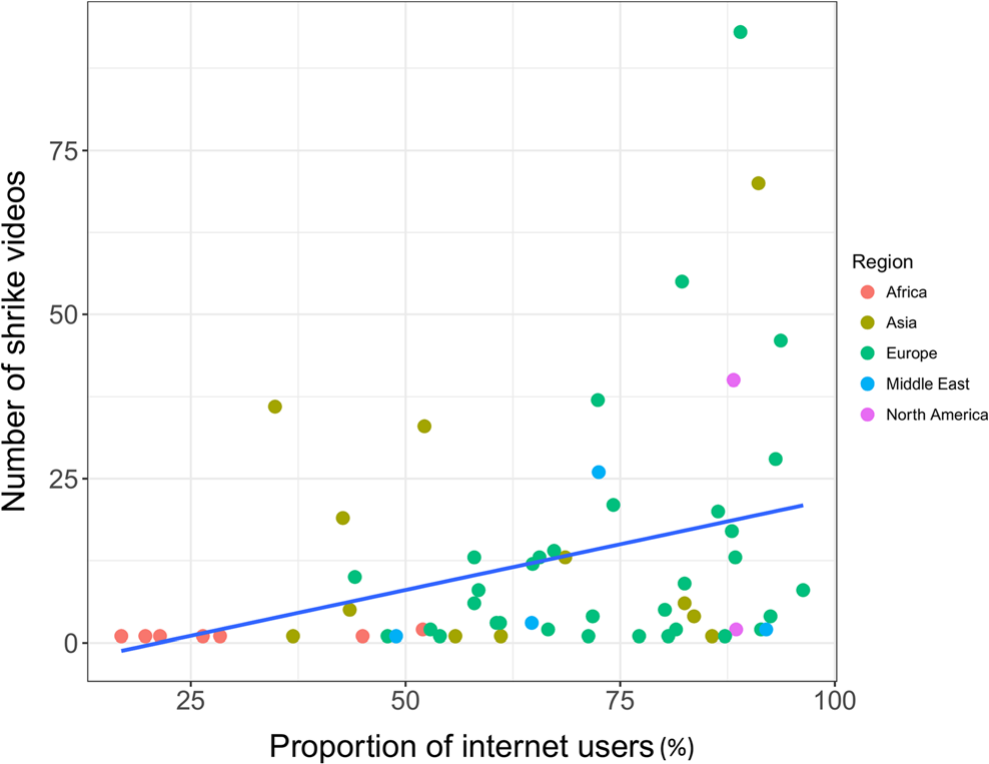
Correlation plot between the proportion of Internet users and the number of shrike videos on YT by country. *Colour points* describe affiliation to world region

On a country level, we found a significant positive correlation between the proportion of Internet users in population and the number of shrike videos on YT (*r* = 0.401, *p* = 0.01, Fig. 2). However, we did not find any correlation between the proportion of smartphone users in the population and the number of shrike videos (*r* = 0.203, *p* = 0.167). The list of countries with the number of shrike videos and proportion of Internet and smartphone users are included in Table S2.

### Shrike behaviour on YT

The most common shrike behaviour on YT included feeding (18.5% of all classified cases), followed by singing (13.6%), comfort behaviour (12.2%), breeding (3.4%), self-defence (2.0%), courtship (0.9%) and interactions with humans (0.7%). Recordings containing other behaviours were represented by 48.6% of all video records. The number of recorded categories of behaviour for one video ranged from 1 to 6 (mean ± SD = 2.37 ± 1.14). The majority of video records came from Europe, Middle Asia, South-East Asia, North America and Northern Africa (Fig. S2). The proportion of recorded behaviour types significantly differs between world regions (*χ* ^2^ = 95.86, df = 32, *p* < 0.001, Table 1).

**Table 1:** Proportion (%) of shrike behaviour on YT videos (*N* = 730) for world regions

Region	Feeding	Breeding	Courtship	Defence	Singing	Comfort behaviour	Relation to human	Other behaviour
Africa	42.86	0	0	0	14.29	7.14	0	35.71
Asia	11.48	1.91	0	0.48	15.31	10.05	0.96	59.81
Europe	19.01	4.37	1.52	0.38	9.89	14.83	0.57	49.43
Middle East	23.26	0	0	0	16.28	23.26	0	37.20
North America	23.81	9.52	0	2.38	19.05	9.52	0	35.72

## Discussion

Shrikes are popular research organisms in behavioural ecology because of their spectacular and unique behaviour of impaling, the ability to coexist in the vicinity of human settlements and relative ease of study because of their conspicuousness in the field (Panov 2011). We have found a strong positive correlation between the number of shrike videos on YT and available scientific literature on shrikes. Unsurprisingly, shrike species of Western Palaearctic and North America are often recorded by the public, and also available publication records on these species are more common than for poorly studied shrike species mainly from tropical regions. This is most probably an effect of the greater number of birdwatchers and ecotourists with a general interest in birds (Glowinski 2008) and also better access to the Internet and recording devices in many northern-hemisphere countries (Fig. S2). However, the lack of correlation between shrike videos and proportion of smartphone users by country is most probably linked to the fact that birdwatchers are using more specialised devices other than smartphone devices such as cameras.

**Fig. 3:**
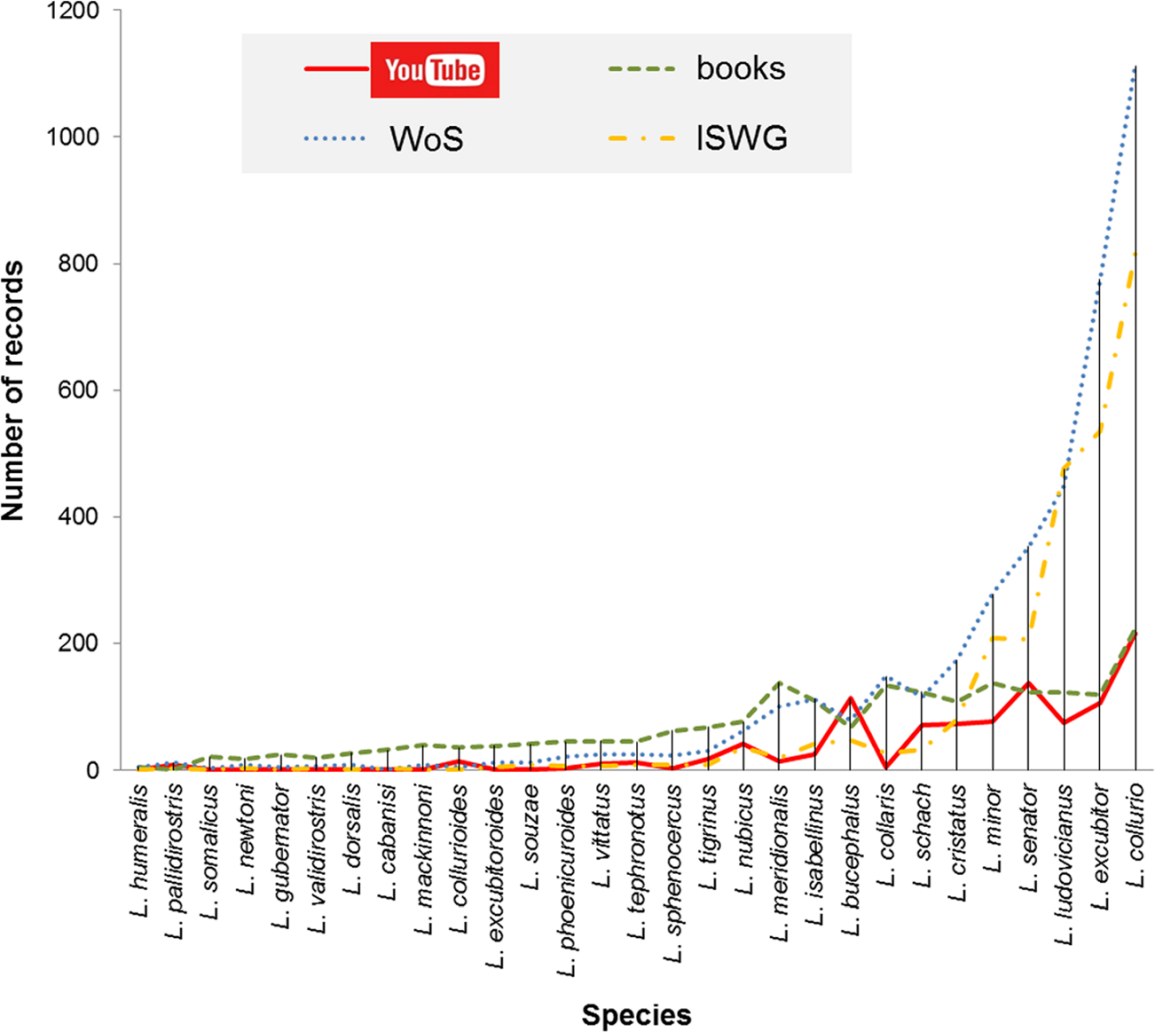
The number of records for 29 shrike species obtained from four different sources: YT, books, WoS and ISWG

Our results also suggest that videos uploaded to YT may provide an alternative source of information about shrike behaviour and ecology especially for species living in the tropics and/or less studied regions where gaps in our knowledge are still very noticeable (Sodhi and Liow 2000). For instance, for some species, we found proportionally more videos on YT than expected from their species-specific publication record (Fig. 3). This is also the case for *L. bucephalus*, a shrike species inhabiting SE Russia, NE China, Korea and all the main islands of the Japanese archipelago. Despite the rather sparse publication record on this species, Japan, China and South Korea have well- and long-established markets for smartphones and other recording devices and widespread Internet upload culture (Mak et al. 2014). These activities may thus help us to fill the gap in information transfer between some world regions and gain also insights into the ecology of local animals.

We also have shown that YT videos contain several categories of behaviour which research is a base stone in behavioural ecology including feeding, breeding and singing (Krebs and Davies 2009). Interestingly, the proportion of some behavioural categories significantly differs between world regions: for instance, videos from Europe only rarely contain singing shrikes while this activity was much common in all other regions. Similarly, shrikes in Africa were very frequently recorded as feeding while birds from Europe and the Middle East were more often involved in comfort behaviour. First, it is possible that our results reflect real differences in the daily activity of shrikes of different populations or species, for instance, in relation to local environmental conditions. For instance, birds from a harsher environment with patchy food distribution are known to spend more time foraging (Brown 1999; Stephens 2008) and higher environmental variability was found to be associated with higher song rates in mockingbirds (Botero et al. 2009). Second, habitat structure can cause some types of behaviour may be more conspicuous and thus are often recorded by local people or tourists. Third, differences in frequency of recorded behaviour between world regions may result from a different perception of animal behaviour by people from different cultures (Lawrence 1986; Morris and Peng 1994; Choi et al. 1999).

It has been suggested that videos are more representative of animal behaviour than the descriptions contained in books or publications. For instance, Panov (2011) describing the courtship behaviour of some shrike species referred the reader to a YT video. Multimedia sources (i.e. picture, animation, simulation and video) offer great potential to learning and teaching (Mayer 2001), and multimedia used in the teaching of some biology aspect are better understood and remembered (Satyaprakasha and Sudhanshu 2014; Karakoyun and Yapici 2016). Moreover, while the two books (Harris and Franklin 2000; Panov 2011) provide vastly more information on the biology of these species, access to these professional books is limited to the professional community. Here, YT videos can be very helpful, providing an alternative source of visualisation of shrike ecology and behaviour for enthusiasts of this group all over the world. Additionally, in some cases, Internet data contained novel localities, not published in the literature to date, including, for instance, some website about terrestrial vertebrate and butterfly observations (https://observation.org), only birds (e.g. http://clanga.com, http://www.audubon.org and http://ebird.org) or Internet ornithology forums (see more in Galán-Díaz et al. 2015). During the past decade, the importance of data shared online including museum and herbaria has rapidly increased (e.g. Graham et al. 2004; Elith et al. 2006). Public Web domains can represent a wealth of readily available information and large amounts of spatially distributed data can be collated for several organisms or groups in tandem; results from such collected data can be in good agreement with fieldwork data with the potential for supplementing less comprehensive and resource-demanding fieldwork (Silvertown 2009; Leighton et al. 2016). However, YT videos must be considered with caution because we have found that such an approach has several limitations: (1) despite the fact that YT videos contain records on interesting and rarely observed shrike behaviour such as courtship or singing, almost half of all videos captured birds in general position including sitting on a perch or looking around and (2) many of the records lack critical information on their geographical locations thus one can only extract very general information on species distribution.

In summary, assessing a subject presented in the social media related to knowledge from scientific literature is an important phenomenon nowadays. YT has many advantages, including free access to videos containing more information than still pictures, hence, not only improving the level of scientific documentation (especially of complex sequential behaviours) but also increasing the availability of such information for a wider population. Moreover, online sources, including YT, may contain new information that has not previously been described in scientific papers or expand science knowledge on poorly studied natural phenomena. For instance, Yosef and McPherson (2016) were able to discern a previously undescribed behaviour in which *Lanius ludovicianus* eviscerate grasshoppers using video footage. Similarly, using classical literature but also Google Images and YT, Mikula et al. (2016) found that bat predation by diurnal birds is a more common interaction than previously documented and online sources are particularly important in less studied regions. This use of social media opens new possibilities to analyse data collected by both scientists and the public (e.g. Leighton et al. 2016), similar to the way that many citizen science projects contribute to our understanding of animal behaviour in the quickly changing or hardly accessible environment. Social media opens up new possibilities not only for nature conservation but also for behavioural research. We think that social media such as YT and Internet access foster a closer relationship between public and scientific researchers.

## Electronic supplementary material

ESM 1 (DOC 823 kb)
