# Supplementary material for: Social media and scientific research are complementary—YouTube and shrikes as a case study
Source: Naturwissenschaften. 2017 May 24;104(5):48. doi: 10.1007/s00114-017-1470-8 (PMC5443854; doi:10.1007/s00114-017-1470-8)
Supplement: Supplementary file 1 — (DOC 823 kb) [file 114_2017_1470_MOESM1_ESM.doc]

Supplementary Materials

**Social media and scientific research are complementary – YouTube and shrikes as a case study**

Łukasz Dylewski^1*^, Peter Mikula^2^, Piotr Tryjanowski^1^, Federico Morelli^3,4^, Reuven Yosef^5^

^1^Institute of Zoology, Poznań University of Life Sciences, Wojska Polskiego 71C, 60-625 Poznań, Poland

^2^Department of Zoology, Faculty of Science, Charles University, Viničná 7, 128 43 Praha 2, Czech Republic

^3^Czech University of Life Sciences Prague, Faculty of Environmental Sciences, Department of Applied Geoinformatics and Spatial Planning, Kamýcká 129, CZ-165 00 Prague 6, Czech Republic

^4^ Faculty of Biological Sciences, University of Zielona Góra, Prof. Szafrana St. 1, PL 65-516 Zielona Góra, Poland

^5^Ben Gurion University of the Negev – Eilat Campus, P. O. Box 272, Eilat 88000, Israel

* Corresponding author

Figure S1–S2

Tables S1–S2

**Figure S1.** The number of YouTube videos recording shrikes per world regions.


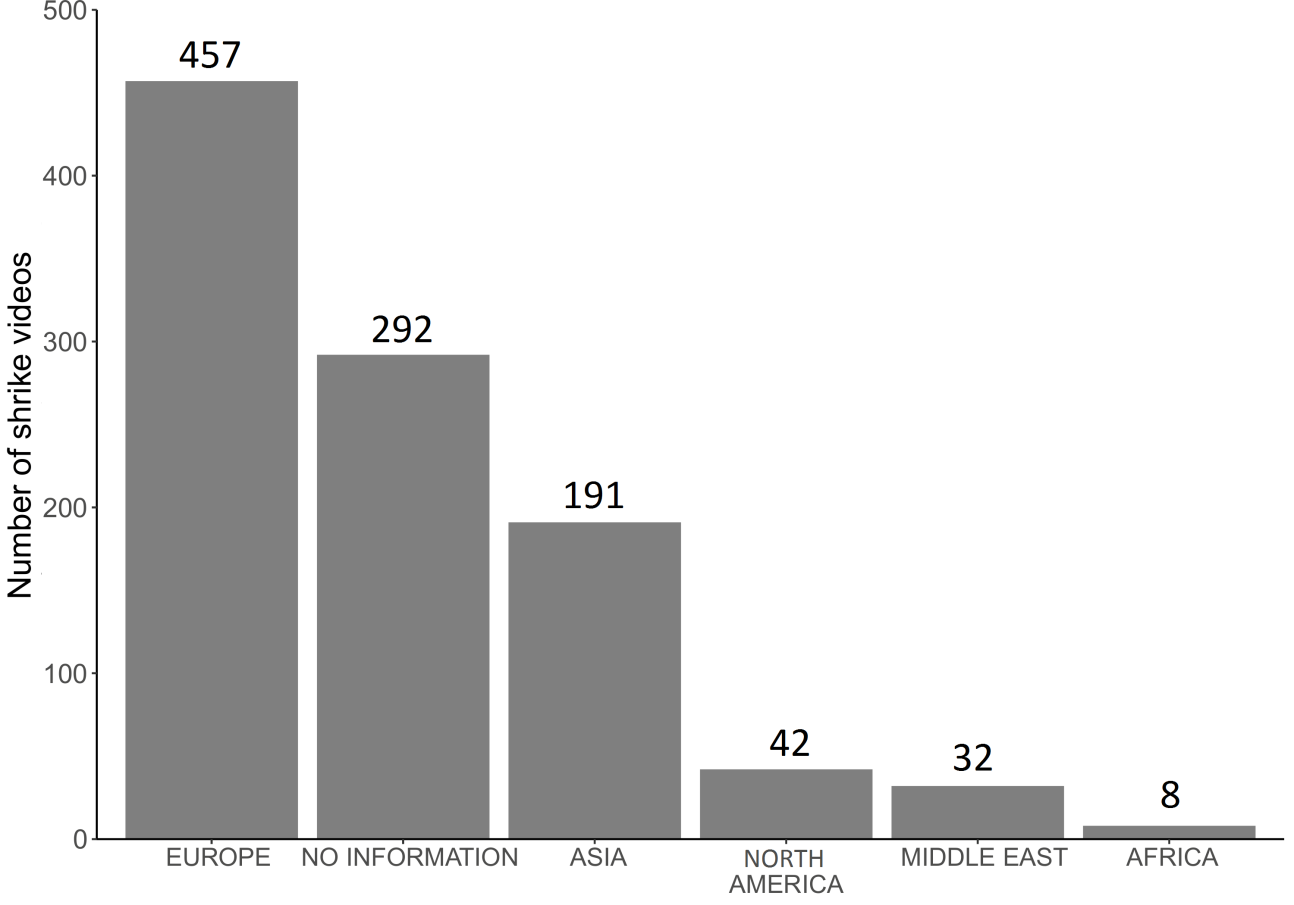


**Figure S2.** Numbers of YT video records for shrikes. The size of the cylinder is proportional to the number of videos per country, and the relative composition inside the chart is related to the category of behaviour captured in the videos.


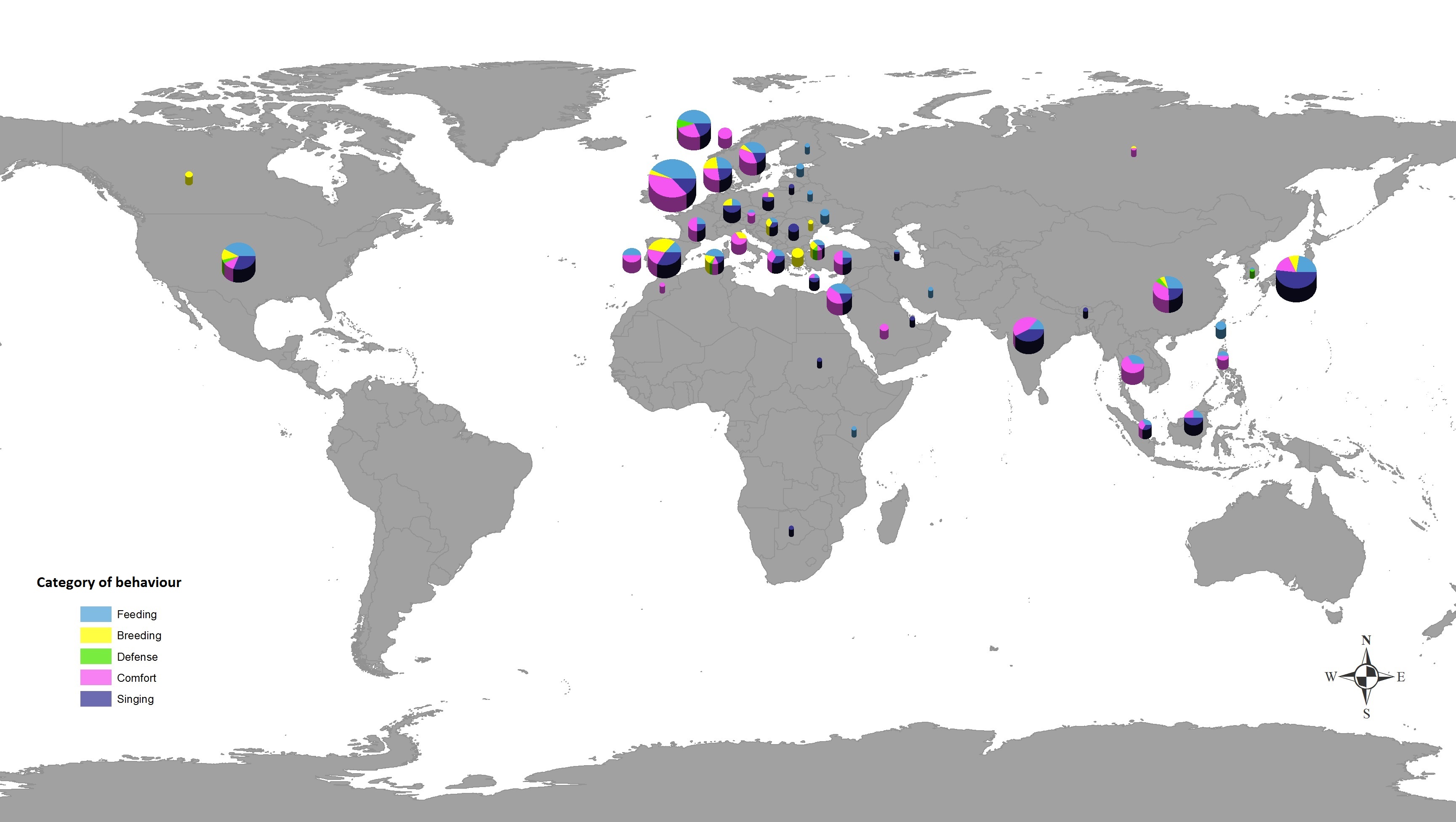


**Table S1.** YouTube video parameters for 24 shrike species.

| Species | Number of videos | Video length (s) | | | Number of views | | | Number of days on YouTube | | | Number of comments | | |
| --- | --- | --- | --- | --- | --- | --- | --- | --- | --- | --- | --- | --- | --- |
|  |  | MEAN | SD | MIN–MAX | MEAN | SD | MIN–MAX | MEAN | SD | MIN–MAX | MEAN | SD | MIN–MAX |
| *Lanius bucephalus* | 113 | 64.22 | 6.032 | 5–330 | 118.26 | 29.004 | 2–2977 | 988.34 | 43.685 | 341–2925 | 0.45 | 0.118 | 0–7 |
| *Lanius cabanisi* | 1 | 8 | – | – | 734 | – | – | 3142 | – | – | 0 | – | – |
| *Lanius collaris* | 5 | 45.4 | 8.738 | 18–68 | 242 | 145.968 | 47–815 | 1502 | 362.613 | 541–2549 | 0 | 0 |  |
| *Lanius collurio* | 216 | 80.77 | 5.519 | 2–572 | 263.71 | 43.468 | 1–7200 | 1075.44 | 44.263 | 198–3476 | 0.73 | 0.297 | 0–59 |
| *Lanius collurioides* | 14 | 46.86 | 9.173 | 9–145 | 69.71 | 22.577 | 13–269 | 937.21 | 139.225 | 274–2124 | 0.14 | 0.097 | 0–1 |
| *Lanius cristatus* | 72 | 63.04 | 7.461 | 10–435 | 325.33 | 64.614 | 3–2987 | 1312.15 | 76.969 | 368–3014 | 0.75 | 0.2 | 0–8 |
| *Lanius excubitor* | 106 | 78.91 | 7.251 | 5–420 | 2395.15 | 1111.461 | 3–94914 | 1382.18 | 80.246 | 161–3288 | 1.49 | 0.642 | 0–66 |
| *Lanius excubitoroides* | 1 | 63 | – | – | 3336 | – | – | 1470 | – | – | 10 | – | – |
| *Lanius isabellinus* | 24 | 89.08 | 19.255 | 4–474 | 265.13 | 41.131 | 20–657 | 1257.88 | 177.581 | 370–3403 | 0.63 | 0.247 | 0–5 |
| *Lanius ludovicianus* | 74 | 65.45 | 6.65 | 5–297 | 1039.41 | 384.946 | 2–26827 | 1233.04 | 94.088 | 285–3388 | 1.43 | 0.431 | 0–22 |
| *Lanius mackinnoni* | 1 | 20 | – | – | 125 | – | – | 765 | – | – | 2 | – | – |
| *Lanius meridionalis* | 14 | 62.64 | 20.601 | 15–320 | 2105.36 | 1686.362 | 2–23949 | 1590.79 | 223.173 | 442–2934 | 3.21 | 2.837 | 0–40 |
| *Lanius minor* | 76 | 72.39 | 9.193 | 7–436 | 198.14 | 49.325 | 5–3582 | 1286.86 | 84.789 | 305–3221 | 0.2 | 0.062 | 0–3 |
| *Lanius nubicus* | 41 | 43.8 | 5.565 | 8–139 | 183.24 | 44.109 | 7–1523 | 1114.63 | 114.36 | 288–3221 | 0.22 | 0.102 | 0–3 |
| *Lanius pallidirostris* | 9 | 65 | 12.808 | 20–137 | 124.44 | 30.604 | 12–252 | 1039 | 130.031 | 431–1627 | 0.44 | 0.294 | 0–2 |
| *Lanius phoenicuroides* | 3 | 77 | 16.523 | 47–104 | 59.67 | 29.294 | 12–113 | 941.33 | 277.529 | 569–1484 | 0.33 | 0.333 | 0–1 |
| *Lanius schach* | 71 | 69.66 | 6.278 | 9–242 | 318.89 | 70.058 | 4–3542 | 1288.58 | 82.173 | 270–3031 | 0.8 | 0.274 | 0–13 |
| *Lanius senator* | 137 | 86.2 | 6.335 | 6–389 | 386.11 | 96.017 | 3–8737 | 1294.64 | 66.223 | 194–3408 | 0.38 | 0.104 | 0–9 |
| *Lanius somalicus* | 1 | 6 | – | – | 58 | – | – | 446 | – | – | 0 | – | – |
| *Lanius sphenocercus* | 3 | 70.33 | 30.024 | 18–122 | 149 | 98.551 | 45–346 | 746.67 | 90.683 | 568–863 | 1 | 1 | 0–3 |
| *Lanius tephronotus* | 12 | 58.83 | 12.92 | 12–150 | 142.5 | 47.566 | 10–507 | 969.58 | 166.806 | 238–2216 | 0 | 0 | 0 |
| *Lanius tigrinus* | 17 | 54.12 | 24.867 | 11–444 | 435.06 | 135.838 | 8–2068 | 1621.88 | 160.592 | 373–2920 | 0.47 | 0.212 | 0–2 |
| *Lanius validirostris* | 1 | 174 | – | – | 1268 | – | – | 2258 | – | – | 1 | – | – |
| *Lanius vittatus* | 10 | 40.8 | 5.479 | 22–76 | 34.4 | 9.758 | 7–90 | 742.9 | 117.234 | 376–1379 | 0.5 | 0.269 | 0–2 |

**Table S2.** The list of 58 country with proportion of internet and smartphone users and number of YouTube videos recording shrikes. Sources: ^1^http://www.internetlivestats.com, ^2^https://www.statista.com, https://www.emarketer.com, http://tech.thaivisa.com/.

| **Region** | **Country** | **Internet users (percent of population)^1^** | **Smartphone users (percent of population)^2^** | **Number of shrike videos** |
| --- | --- | --- | --- | --- |
| AFRICA | Algeria | 19.7 | 20.0 | 1 |
| AFRICA | Botswana | 21.4 | – | 1 |
| AFRICA | Gambie | 16.9 | – | 1 |
| AFRICA | Ghana | 28.4 | 21.0 | 1 |
| AFRICA | Kenya | 45.0 | 26.0 | 1 |
| AFRICA | South Africa | 52.0 | 37.0 | 2 |
| AFRICA | Sudan | 26.4 | – | 1 |
| N. AMERICA | Canada | 88.5 | 67.0 | 2 |
| N. AMERICA | USA | 88.2 | 72.0 | 40 |
| ASIA | Azerbaijan | 61.1 | – | 1 |
| ASIA | Bhutan | 36.9 | – | 1 |
| ASIA | China | 52.2 | 58.0 | 33 |
| ASIA | India | 34.8 | 17.0 | 36 |
| ASIA | Japan | 91.1 | 39.0 | 70 |
| ASIA | Kazakhstan | 55.8 | 15.7 | 1 |
| ASIA | Malaysia | 68.6 | 65.0 | 13 |
| ASIA | Philippines | 43.5 | 22.0 | 5 |
| ASIA | Singapore | 82.5 | 71.7 | 6 |
| ASIA | South Korea | 85.7 | 88.0 | 1 |
| ASIA | Taiwan | 83.6 | 51.0 | 4 |
| ASIA | Thailand | 42.7 | 43.0 | 19 |
| EUROPE | Austria | 81.5 | 63.0 | 2 |
| EUROPE | Belarus | 61.0 | – | 3 |
| EUROPE | Bulgaria | 58.5 | 43.0 | 8 |
| EUROPE | Croatia | 74.2 | 51.0 | 21 |
| EUROPE | Cyprus | 71.8 | 52.0 | 4 |
| EUROPE | Czech Republic | 88.4 | 50.0 | 13 |
| EUROPE | Denmark | 96.3 | 59.0 | 8 |
| EUROPE | Estonia | 91.4 | 51.0 | 2 |
| EUROPE | Finland | 92.5 | 57.0 | 4 |
| EUROPE | France | 86.4 | 49.0 | 20 |
| EUROPE | Georgia | 52.9 | – | 2 |
| EUROPE | Germany | 88.0 | 60.0 | 17 |
| EUROPE | Gibraltar | 66.6 | – | 2 |
| EUROPE | Greece | 64.8 | 37.0 | 12 |
| EUROPE | Hungary | 80.2 | 47.0 | 5 |
| EUROPE | Ireland | 80.6 | 57.0 | 1 |
| EUROPE | Italy | 65.6 | 60.0 | 13 |
| EUROPE | Lithuania | 77.2 | 48.5 | 1 |
| EUROPE | Morocco | 60.6 | – | 3 |
| EUROPE | Moldova | 47.9 | – | 1 |
| EUROPE | Netherlands | 93.7 | 70.0 | 46 |
| EUROPE | Poland | 72.4 | 41.0 | 37 |
| EUROPE | Portugal | 67.3 | 52.4 | 14 |
| EUROPE | Romania | 58.0 | 39.7 | 6 |
| EUROPE | Russia | 71.3 | 45.0 | 1 |
| EUROPE | Serbia | 54.0 | 42.0 | 1 |
| EUROPE | Slovakia | 82.5 | 51.2 | 9 |
| EUROPE | Spain | 82.2 | 65.0 | 55 |
| EUROPE | Sweden | 93.1 | 73.0 | 28 |
| EUROPE | Switzerland | 87.2 | 69.0 | 1 |
| EUROPE | Turkey | 58.0 | 59.0 | 13 |
| EUROPE | United Kingdom | 89.0 | 68.0 | 93 |
| EUROPE | Ukraine | 44.1 | 27.0 | 10 |
| MIDDLE EAST | Iran | 48.9 | 52.0 | 1 |
| MIDDLE EAST | Israel | 72.5 | 74.0 | 26 |
| MIDDLE EAST | Qatar | 92.0 | 75.0 | 2 |
| MIDDLE EAST | Saudi Arabia | 64.7 | 72.8 | 3 |
